# Effects of Amyloid Beta (Aβ) Oligomers on Blood–Brain Barrier Using a 3D Microfluidic Vasculature-on-a-Chip Model

**DOI:** 10.3390/app14093917

**Published:** 2024-05-04

**Authors:** Samuel Chidiebere Uzoechi, Boyce Edwin Collins, Cody Joseph Badeaux, Yan Li, Sang Su Kwak, Doo Yeon Kim, Daniel Todd Laskowitz, Jin-Moo Lee, Yeoheung Yun

**Affiliations:** 1 Department of Chemical, Biological, and Bioengineering, North Carolina Agricultural and Technical State University, Greensboro, NC 27411, USA;; 2 Department of Biomedical Engineering, Federal University of Technology, PMB 1526, Owerri 460114, Nigeria; 3 Chemical & Biomedical Engineering, College of Engineering, Florida State University, Tallahassee, FL 32310, USA;; 4 Genetics and Aging Research Unit, Mass General Hospital, Harvard Medical School, 114 16th Street, Charlestown, MA 02129, USA;; 5 Neurosurgery, Anesthesiology & Neurobiology, Duke University Medical Center, Durham, NC 27710, USA;; 6 Department of Neurology, Washington University School of Medicine, St. Louis, MO 63110, USA;

**Keywords:** blood–brain barrier, microfluidic, amyloid beta, Alzheimer’s Disease (AD)

## Abstract

The disruption of the blood–brain barrier (BBB) in Alzheimer’s Disease (AD) is largely influenced by amyloid beta (Aβ). In this study, we developed a high-throughput microfluidic BBB model devoid of a physical membrane, featuring endothelial cells interacting with an extracellular matrix (ECM). This paper focuses on the impact of varying concentrations of Aβ_1–42_ oligomers on BBB dysfunction by treating them in the luminal. Our findings reveal a pronounced accumulation of Aβ_1–42_ oligomers at the BBB, resulting in the disruption of tight junctions and subsequent leakage evidenced by a barrier integrity assay. Additionally, cytotoxicity assessments indicate a concentration-dependent increase in cell death in response to Aβ_1–42_ oligomers (LC50 ~ 1 μM). This study underscores the utility of our membrane-free vascular chip in elucidating the dysfunction induced by Aβ with respect to the BBB.

## 1. Introduction

The blood–brain barrier (BBB) is necessary for normal brain function and maintains the central nervous system (CNS) homeostatic environment. Specialized endothelial cells and supporting cells such as neurons, oligodendrocytes, microglia, astrocytes, and pericytes make up the blood–brain barrier (BBB) [1,2]. Adherens junctions and tight junctions, along with a few other unique transport mechanisms, allow the blood–brain barrier (BBB) to regulate the entry of substances into the brain [3,4]. The BBB’s barrier qualities, however, also make it more difficult to treat CNS illnesses, since they prevent many large- and small-molecule medications from getting into the brain in amounts sufficient to cause a therapeutic reaction [5]. With respect to Alzheimer’s disease (AD), studies show that Aβ_1–42_ plays a central role in Aβ accumulation and plaque formation in the brain [6,7]. The blood–brain barrier (BBB) plays a crucial role in the regulation of Aβ levels in the brain, and its dysfunction is closely related to the accumulation of Aβ caused by Alzheimer’s disease (AD) [6,7]. Understanding vascular contributions to cognitive impairment and dementia (VCID) is significant due to the cognitive decline resulting from damage to the brain function [8]. Another aspect of AD-related emerging issues is cerebral amyloid angiopathy (CAA). CAA is a type of cerebrovascular disorder characterized by the accumulation of Aβ within the leptomeninges and small-to-medium-sized cerebral blood vessels [9]. CAA is associated with cognitive dysfunction, intracerebral hemorrhage (ICH), and microinfarcts. CAA is tightly linked with FDA-approved anti-Aβ immunotherapy as a side effect [10]. The main receptors for Aβ transport across the BBB from brain to blood and blood to brain are low-density lipoprotein receptor-related protein-1 (LRP1) and receptor for advanced glycation end products (RAGE), respectively [11,12]. Tight junction (TJ) proteins such as ZO-1, claudin-5, and occludin are part of the components of the BBB and are considered, in part, important indicators of the morphological changes in the BBB [13]. Apart from the molecular basis of AD, this model can be used to model other neurological disorders and neurodegenerative diseases [14].

Although whole-organism drug distribution can be studied using in vivo models, their use for BBB-penetrant antibody screening is limited due to the intricacy of this process and the reduced throughput of these investigations. Because of this, the field’s in vivo research is supplemented with quicker and easier in vitro models, such as the transwell technique [15–17] and several on-a-chip technologies [18–20]. Even though in vitro BBB modeling has advanced significantly in recent years, a model that combines physiologically appropriate circumstances such as flow, coculture, and the absence of artificial membranes with quick, high-throughput readouts is still required. To address some of the key limitations of the current models, we generated a 3D bioengineered microfluidic membrane-free BBB against ECM using a phase guide method, a geometric feature that acts as a pressure barrier due to meniscus pinning. Here, we studied Aβ trafficking through the BBB using the two-lane perfusion-based microfluidic device. This perfusion-based microfluidic device would offer numerous advantages, including reproducibility, reduced sample volumes and cost, precise control of media flow and reagent delivery, and the ability to regulate the spatiotemporal parameters of the brain microenvironment.

## 2. Materials and Methods

### 2.1. Cell Culture

Primary human umbilical vein endothelial cells (HUVECs-C2519AS) were purchased from Lonza (Basel, Switzerland) [21]. Endothelial cells were expanded in T-75 flasks (Nunc^™^ EasyFlask, Sigma F7552 (Sigma-Aldrich, St. Louis, MI, USA)) and used for four-to-six passages and cultured in endothelial cell growth medium-2 (EGM^™^-2) and BulletKit^™^ (Lonza, Basel, Switzerland, CC-3162), with media change occurring every 3 days [22]. All cells were cultured in a humidified incubator at 37 °C and 5% CO_2_ to allow for optimal cell growth and differentiation.

### 2.2. Microfluidic Cell Culture

**Figure 1.**
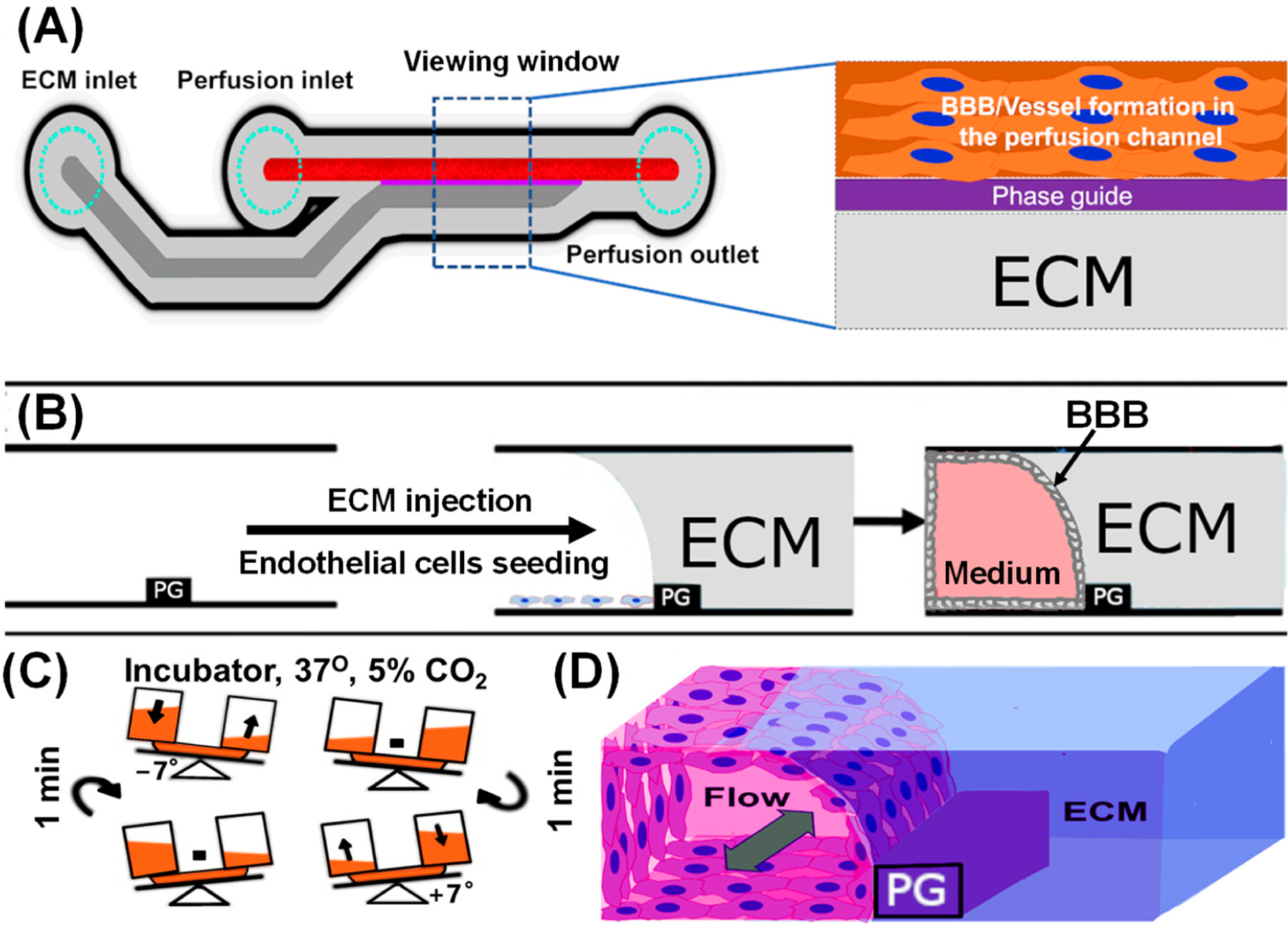
The schematic diagram for the construction of the 3D BBB system in the microfluidic device. (**A**) The schematic structure of a microfluidic chip and the representative design depicting the observation window. (**B**) Experimental procedure for the construction of a 3D neurovascular/brain compartment. (**C**) The incubation of a microfluidic device in a dynamic perfusion that allows bidirectional fluid flow. (**D**) Schematic illustration of the expected 3D reconstruction of the endothelial-cell-based vascular barrier (tunable perfusion).

The membrane-free blood–brain barrier (BBB) was constructed using a microfluidic device. A two-lane microfluidic OrganoPlate with 96 vascular channels and with dimensions 400 μm × 220 μm (w × h) and a phase guide of dimensions 100 μm × 55 μm (w × h) (MIMETAS BV, Gaithersburg, MD, USA) were used to engineer the BBB-like construct [23]. Figure 1 shows the process of BBB tissue construction. The channels were injected with 2 μL of extracellular matrix (ECM) prepared at the ratio of 8:1:1 of type I (Corning, Collagen I Rat Tail, 9.48 mg/mL), 1 M HEPES (ThermoFisher, Norristown, PA, USA, 15630–080), and 37 g/L NaHCO_3_, and the OrganoPlate was incubated for 15 min at 37 °C and 5% CO_2_ to allow for ECM to polymerize [23]. To prevent the polymerized ECM from drying out, 50 μL of HBSS (ThermoFisher, 14175–079) was added to the gel inlet. Subsequently, p4 endothelial cells were harvested with TrypLE^™^ Express (ThermoFisher, 12605010). HUVECs were seeded in microfluidics at the density of 10,000 cells/μL. A total of 2 μL of endothelial cell suspension was seeded in the medium inlet (perfusion channel or blood lane), 50 μL of endothelial cell complete media was added to the top medium inlet, and HBSS was aspirated from the gel inlet. The plate was incubated at its side in the plate stand for 6 h to allow the cells on the top channel to settle against the ECM and attach [23,24]. Afterward, 50 μL of endothelial cell complete media was added to the perfusion media outlet and placed on the interval rocker switching between a +7° and a −7° angle of inclination every 8 min, enabling bidirectional fluid flow [23]. The cells were cultured for 5 days at 37 °C and 5% CO_2_ to form BBB against ECM before being treated with Aβ_1–42_ at different concentrations. The endothelial cell medium on both perfusion inlet and outlet was refreshed every two days with 50 μL each.

### 2.3. Amyloid Beta 1–42 (Aβ_1–42_) Oligomer Preparation and Treatment

Biotinyl–Amyloid β Protein (Aβ_1–42_) was purchased (Bachem, Bubendorf, Switzerland, 4038795) and stored in desiccated containers at −80 °C. Before use, the peptide was allowed to equilibrate to room temperature for at least 30 min to avoid condensation upon opening the vial [25]. A 1 mM of Biotinyl–Amyloid β Protein (Aβ_1–42_) treatment solution was prepared in the fume hood by dissolving 0.5 mg of Aβ_1–42_ in 105.5 μL of 100% 1,1,1,3,3,3-Hexafluoro-2-propanol (HFIP) (ThermoFisher, 147541000) [25,26]. The clear solution containing 1 mM of dissolved Aβ_1–42_ was then aliquoted in 0.6 μL low-retention microcentrifuge tubes (ThermoFisher, 3449). The samples were left in the fume hood overnight to allow HFIP to evaporate and were left 1 h in the desiccator to promote evaporation of any remaining HFIP. Then, the subsequent clear Aβ_1–42_ film was resuspended in sterile DMSO (Sigma-Aldrich, 08418) by gently scraping up and down until the peptide film was completely dissolved [26]. The peptide solution was mildly vortexed for 30 s, followed by 5 min of sonication in an ultrasonic bath cleaner (Branson 2510). Twenty-four hours before treatment, Aβ_1–42_ solutions were diluted in EGM-2 medium to the final concentrations of 10 μM, 20 μM, and 30 μM, respectively. A 5-day culture of endothelial cells in the microfluidic device was treated with Aβ_1–42_ solutions for 48 h. The following design was used throughout the experiments: (1) BBB-No Aβ (BBB without Aβ), (2) ECM Only + 20 μM (Aβ without BBB), and (3) 10 μM, 20 μM, and 30 μM (Aβ with BBB). It is important to note that ECM was injected into the gel channel in all conditions.

### 2.4. Barrier Integrity Assay

The barrier integrity assay was performed to measure the barrier function and lumen formation of endothelial vessels grown in the microfluidic device. Perfusion channels were washed once for 5 min with 25 μL EGM-2 complete media to confirm the appropriate flow profile during successive barrier integrity assays [23]. Afterward, all media were aspirated from the chips. A fluorescein isothiocyanate FITC-dextran (MW: 40 kDa; Chondrex) solution was made by dissolving 0.5 mg/mL solution in complete EGM-2 media. Then, 20 μL of EGM-2 media without fluorescent dye was added to the gel inlet (basal side of the chip). A total of 40 μL and 30 μL of FITC solution was added to the media inlet and outlet which contained endothelial microvessels, and the image acquisition was started. Image acquisition was performed on an EVOS M5000 multichannel fluorescence microscope coupled with a stage incubator using the time-lapse function (10-min, 20-s intervals). The apparent permeability was quantified using ImageJ (Version 1.54i, March 2024) [27]. Data were plotted using GraphPad Prism 8.0 (GraphPad Software, San Diego, CA, USA). We used the following formula to get the apparent permeability (***P***_*app*_) value [28]:

$$P_{\text{app}}=\frac{\Delta C_{\text{reciver}}\times V_{\text{reciver}}}{\Delta t\times A_{\text{barrier}}\times C_{\text{donor}}}\left(\frac{\text{cm}}{\text{s}}\right)$$

The fluorescence intensity measured in the ECM channel between ***t*** = 0 and ***t*** = 10 min is represented by Δ***C***_***receiver***_. The volume of the measured region in the ECM channel (h × w × l) is represented by ***V***_***receiver***_. Δ***t*** is the duration between start and finish (10 min). The surface of the ECM interface with the medium channel is represented by ***A***_***barrier***_, and the fluorescence intensity measured in the top perfusion channel is represented by ***C***_***donor***_. This formula assumes that the curve is linear, often maintained when the receiver’s concentration is less than 10% of the donor’s [28].

### 2.5. Immunocytochemistry

Unless otherwise stated, all steps were performed at room temperature on a rocking platform. Briefly, a 3D microfluidic model of the endothelial cell was fixed with 4% PFA (ThermoFisher, J19943-K2) for 15 min, followed by permeabilization with 0.3% Triton X-100 (ThermoFisher, EP151–100) in PBS for 10 min. Then the samples were washed with 4% FBS (Atlanta Biological, Flowery Branch, GA, USA, S11050) in PBS for 5 min. Afterward, the samples were blocked with 2% FBS (Atlanta Biological, S11050), 2% BSA (Sigma-Aldrich, AB412), and 0.1% Tween 20 (Sigma Aldrich, P9416) in PBS (1× Corning, Corning, NY, USA, 21–040-CV) for 45 min. Respectively, 10 μL of solution of Streptavidin conjugated to secondary antibody at 1:100 (AF 594, Invitrogen, Carlsbad, CA, USA, S11227) and ZO-1 conjugated to secondary antibody at 1:100 (AF488; Invitrogen, 339188) were added to the media inlet and outlet and incubated overnight at 4 °C on a rocking platform. Afterward, the plate was washed and 20 μL of Hoechst 1:500 (Invitrogen, Waltham, MA, USA, H3570) was added to the media inlet and outlet overnight. Data were acquired and processed using a two-photon confocal microscope (ZEISS Multiphoton LSM 710) equipped with ZEN Blue software (Version 3.6, Carl Zeiss Microscopy GmbH, Oberkochen, Germany).

### 2.6. Cell Viability Assay

First, the culture medium was aspirated, and the cells were washed with PBS (1X Corning, 21–040-CV; 50 μL for inlet and outlet, respectively) on a rocker. Cell viability was determined by staining the channels with a two-probe solution containing 4 mM of ethidium homodimer-1 (EthD-1, Invitrogen, E1169) in PBS and 2.4 mM of calcein AM (Invitrogen, L3224) [21]. A total of 50 μL and 10 μL of the stain solution were added to the perfusion/media inlet and outlet and rocked for 30 min. The channels were washed with PBS for 1 min while rocking (50 μL into the media inlet and outlet, respectively). A total of 50 μL of PBS was added to the inlets and outlets, and the samples were imaged using ImageXpress Micro Confocal (Molecular Devices).

### 2.7. Statistical Analysis

Statistical analyses were performed using Prism GraphPad software (version 8.0, GraphPad Software, San Diego, CA, USA). Descriptive statistics such as mean, standard deviation, and frequency were calculated for all variables of interest. One-way analysis of Variance (ANOVA) was conducted as appropriate. Statistical significance was considered at a 99.9% confidence interval (*p* < 0.001) unless otherwise stated. The results of the statistical analyses are reported with appropriate measures of effect size and confidence intervals. Perfusion rates were quantified by analyzing images with ImageJ software.

## 3. Results

### 3.1. Microfluidic 3D BBB-on-a-Chip

**Figure 2.**
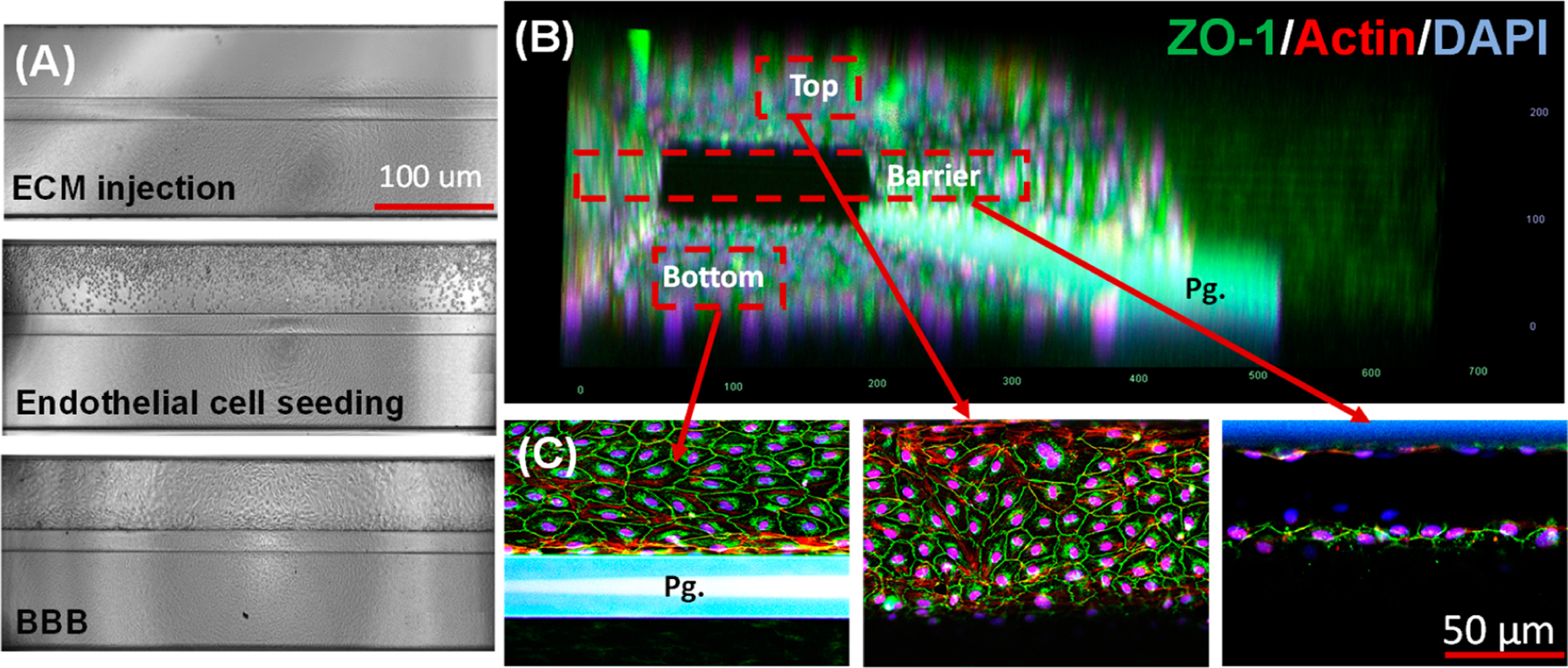
Perfused microvessels of endothelial cells in the microfluidic device. (**A**) The representative phase-contrast images of the BBB formation, (**B**) 3D construction of a confocal image showing a perfused endothelial microvessel with the lumen, the phase guide, and the ECM channel. (**C**) Immunostaining of an endothelial microvessel expressing the tight junction marker, ZO-1 (green), red-phalloidin stain for actin filaments, and DAPI (blue) stain for nuclei. All the images were acquired from the 8-day-old culture using a two-photon confocal microscope (ZEISS Multiphoton LSM 710) equipped with ZEN Blue software (Version 3.6, Carl Zeiss Microscopy GmbH).

Figure 2A shows bright-field images of the BBB tissue construction process with (1) ECM injection, (2) endothelial cells (ECs) seeding against ECM, and (3) membrane-free BBB formation. Using capillary pressure barriers known as phase guides, we first loaded the ECM into the brain lane (or ECM channel) and allowed the ECM to polymerize in the incubator [21]. The endothelial cells were seeded on the blood lane (or perfusion channel) against the ECM on the brain lane (Figure 2A). In 5–7 days, endothelial cells created a confluent monolayer after adhering steadily to the ECM, and then they produced a tubular structure (Figure 2B). A perfusion rocker was used to maintain the plate shear stress and fluidic flow (Figure 1C). The final BBB tissue construct was used for different functional assays, including barrier integrity, amyloid beta trafficking, migration, and cell viability.

Figure 2B shows a 3D reconstruction of a confocal image showing endothelial tubule formation featuring a top, middle, and bottom. Endothelial cell immunostaining showed the robust expression of ZO-1 (green) in cell margins which is characteristic of the BBB (Figure 2C). In addition to ZO-1 staining, we observed a distribution of actin filament (red) throughout the vascular bed and especially along the barrier. Figure 2C also shows the middle section of the BBB, showing the formation of a tight junction against the ECM, maintaining the barrier function without any physical membrane.

### 3.2. Barrier Formation Characterization

**Figure 3.**
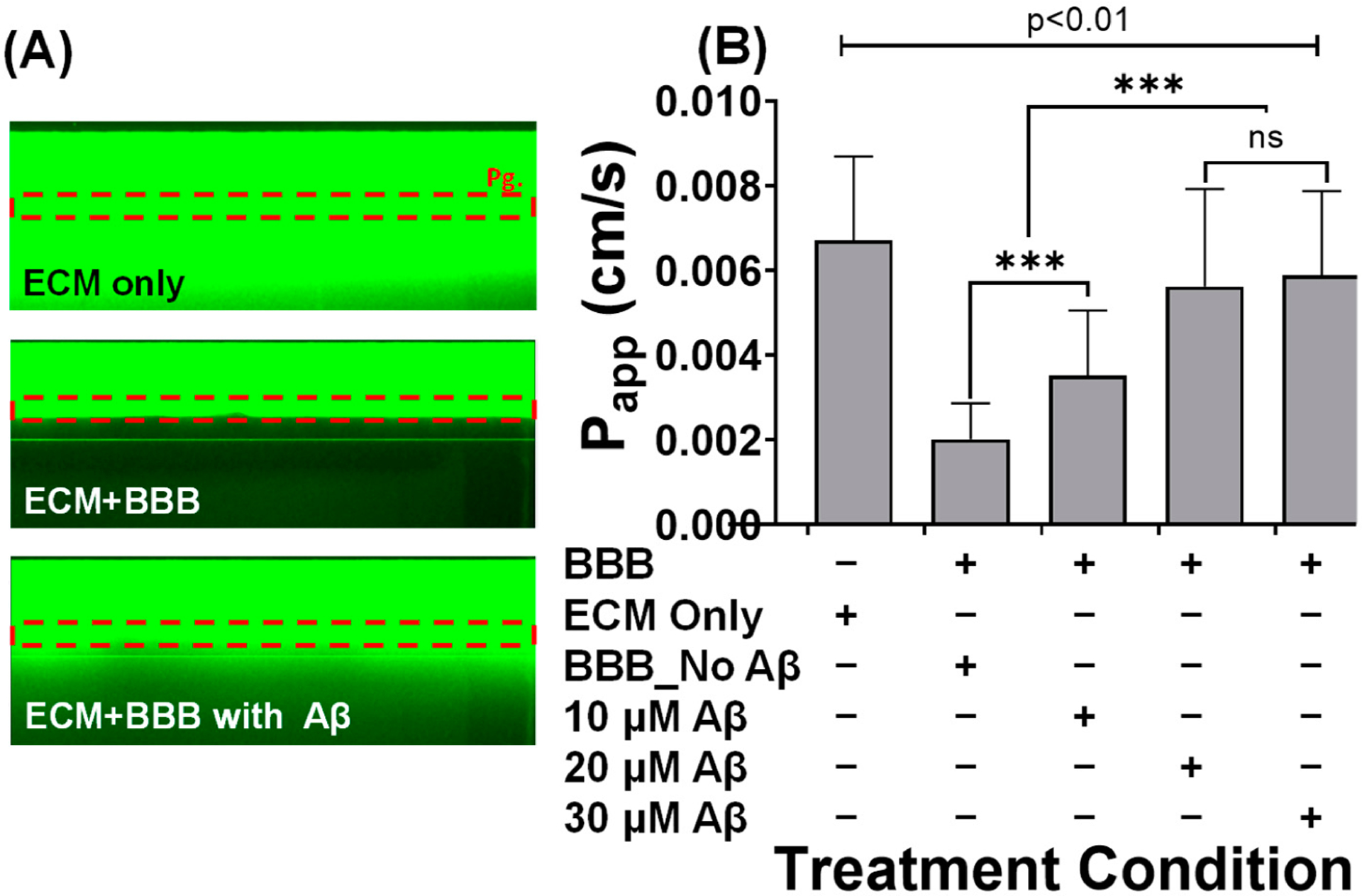
Characterization of the barrier formation in microfluidics. (**A**) Distributions of FITC dextran in the endothelial microvessel. Images were acquired in the absence of the BBB (ECM only, top), a leak-tight barrier (BBB without Aβ_1–42_, middle), and a leaky barrier (BBB with Aβ_1–42_, bottom). (**B**) Apparent permeability for assessing barrier function (for FITC-dextran, 40 kDa) at different time points for endothelial cells cultured under perfusion in the microfluidic device. Error bars show the standard deviation of the mean. The value represents the mean ± SD of two independent experiments with two replicates for each treatment condition. ns not significant and *** indicates *p* < 0.0001 compared with control (BBB_No Aβ) unless otherwise stated.

The barrier integrity of the BBB in the microfluidic device was determined by perfusing the media channels with fluorescein isothiocyanate FITC-dextran (MW: 40 kDa). The permeability of the dye from the endothelial lumen into the adjacent ECM channels was monitored by the acquisition of fluorescent images using the time-lapse function with the stage incubator (Figure 3A). In the chips with an engineered BBB, but without the induction of Aβ_1–42_ solution, a significant amount of the dye was found within the lumen (Figure 3A), and the average apparent permeability (***P***_*app*_) was 2.01 × 10^−3^ (Figure 3B). The chips without BBB (ECM only) and chips with BBB but induced with the Aβ_1–42_ solution demonstrated significant diffusion of the dye into the adjacent ECM channel (Figure 3A), and the ***P***_*app*_ values were 6.72 × 10^−3^, 3.52 × 10^−3^, 5.62 × 10^−3^, and 5.89 × 10^−3^, respectively (Figure 3B).

When the ***P***_*app*_ values in the BBB with or without Aβ_1–42_ are compared, it is clear that Aβ induced a 1.75-fold (10 μM), 2.79-fold (20 μM), and 2.93-fold (30 μM) increase in FITC permeability in the ECM (Figure 3B). However, ECM only showed a 3.34-fold increase in ***P***_*app*_ value when compared with the BBB without Aβ_1–42_ induction. When ECM only is compared with the highest concentration of Aβ_1–42_ (30 μM), the permeability can be seen to have experienced a 1.14-fold increase. Although the Aβ_1–42_ caused damage to the BBB, the presence of endothelial cells provided an additional barrier (Figure 3B).

### 3.3. Aβ_1–42_ Treatment and Associated Tight Junction Behavior

**Figure 4.**
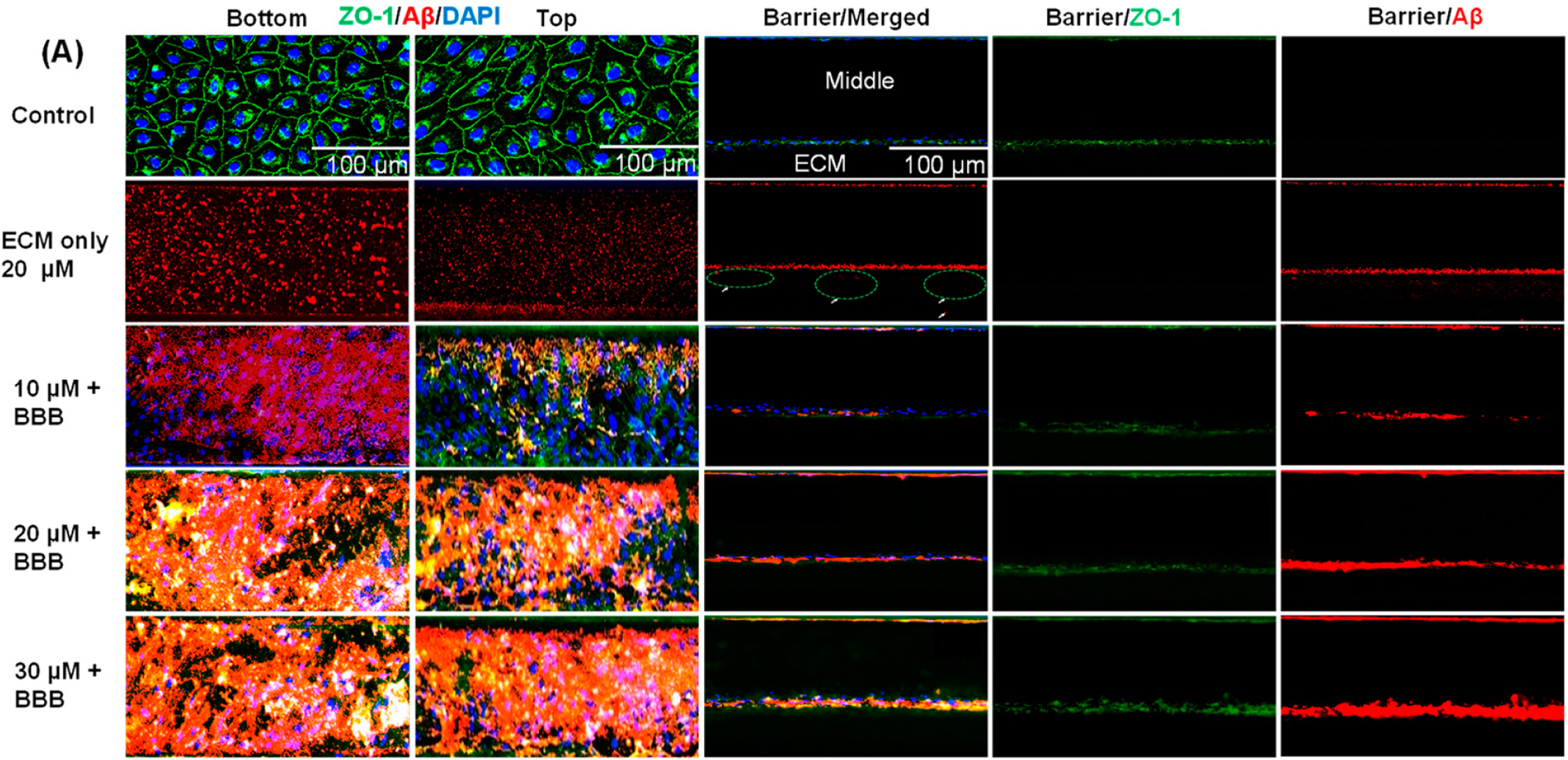

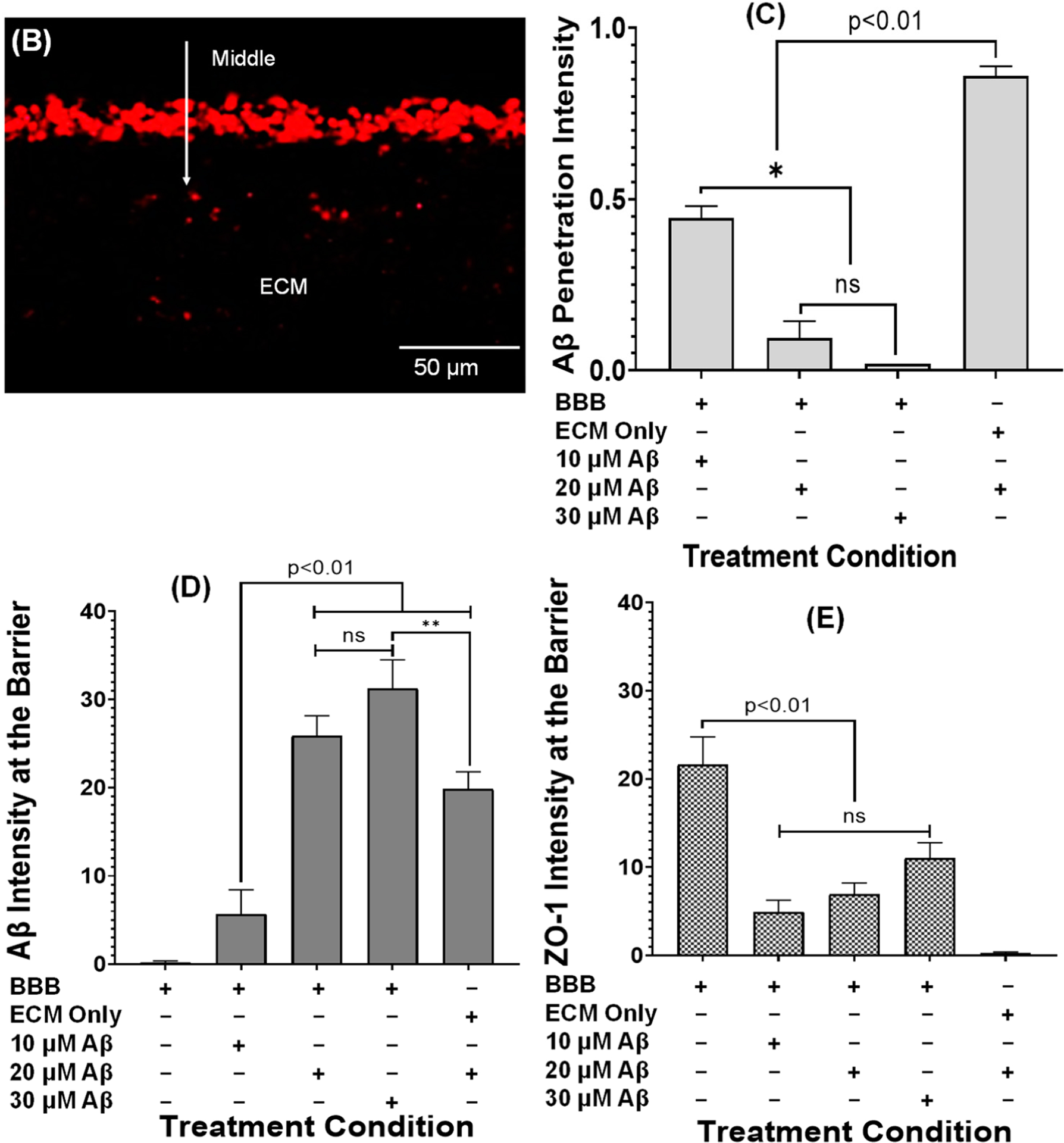
(**A**) Representative immunofluorescence images of 5 days of culture in a microfluidic device that were exposed to nothing (control) or Aβ1–42 (10 μM, 20 μM, and 30 μM) for 48 h are shown (n = 2). They display the blood lane stained images of tight junction (ZO-1, green), amyloid beta (Aβ1–42_,_ red), and nucleus (Hoechst, blue). The arrowheads indicate Aβ1–42 that has penetrated the ECM in a chip without a barrier (Figure A-, ECM only 20 μM). (**B**) Representative image depicting penetration of Aβ_1–42_ into the ECM channel in the absence of an endothelial barrier (ECM only 20 μM). (**C**) Quantification of the average intensity of Aβ_1–42_ penetrated the ECM channel after 48 h. ECM only treated with 20 μM of Aβ_1–42_ is the control for this figure. (**D**,**E**) Aβ_1–42_ and ZO-1 quantification at the barrier at different concentrations using the same image threshold. The value represents the mean ± SD from two independent experiments with two replicates for each treatment condition. ns not significant, * indicates *p* < 0.05 and ** indicates *p* < 0.01 compared with one another unless otherwise stated. All conditions were compared to one another.

It is important to note that, in this study, we analyzed only ZO-1 expression. While claudin-5 and occludin are critical components of tight junctions, ZO-1’s multifaceted role as a scaffolding protein, a regulator of barrier function, and a mediator of signaling pathways makes it particularly important for tight junction expression. Its interactions with other tight junction proteins contribute to the overall integrity and functionality of tight junction complexes. We treated the BBB layer with four concentrations (0 μM, 10 μM, 20 μM, and 30 μM) of Aβ_1–42_ to evaluate their impact on the BBB; we also treated the ECM-only channel without the BBB layer with a 20 μM Aβ_1–42_ concentration to quantify the penetration of Aβ into the brain lane (Figure 4). Endothelial cells challenged with different concentrations of Aβ_1–42_ oligomer solutions for 48 h displayed an altered plasma membrane morphology of ZO-1 with non-specific staining for Aβ_1–42_ (Figure 4A). Quantification of Aβ_1–42_ migration and BBB formation and disruption were based on immunocytochemistry staining (Figure 4). The results were compared with the control (BBB model with no exposure to Aβ_1–42_) (Figure 4). When the chip with ECM only was treated with 20 μM of Aβ_1–42_, there was an increased Aβ_1–42_ intensity in the ECM channels in comparison with the 10 μM (*p* = 0.0432), 20 μM (*p* = 0.0331), and 30 μM (*p* = 0.0219) Aβ_1–42_ treatment (Figure 4B,C). The lowest Aβ_1–42_ intensity in the ECM channels with BBB was found under the 30 μM condition in comparison with the 20 μM (*p* = 0.4852) and 10 μM (*p* = 0.0412) treatments (Figure 4B,C). As expected, we observed that Aβ is associated with a higher ECM penetration if there is no BBB in the chip (ECM only) (Figure 4C).

In all conditions, Aβ_1–42_ and ZO-1 expression at the barrier were quantified. The increase in Aβ_1–42_ intensity on the barrier of the endothelial tubule was associated with an increase in the concentration gradient of Aβ_1–42_ (Figure 4D). Aβ_1–42_ deposition on the barrier was compared, and the 30 μM treatment displayed the highest Aβ_1–42_ intensity in comparison with the 20 μM (*p* = 0.2747), 10 μM (*p* = 0.0006), ECM only + 20 μM (*p* = 0.0229), and BBB-No Aβ conditions (*p* = 0.0002) (Figure 4D). There was no significant difference in Aβ_1–42_ intensity between ECM only treated with Aβ 20 μM and the BBB layer treated with Aβ 20 μM (*p* = 2107). Our analysis shows that the ZO-1 intensity obtained at the barrier was higher in the BBB-No Aβ treatment in comparison with the 10 μM Aβ (*p* = 0.0013), 20 μM Aβ (*p* = 0.0024), and 30 μM Aβ (*p* = 0.0104) conditions (Figure 4E). ZO-1 staining did not reveal any significant differences between the 10 μM and 20 μM (*p* = 0.0921) treatmenets or between the 10 μM and 30 μM (*p* = 0.2944) treatments (Figure 4E).

### 3.4. Effect of Aβ_1–42_ on Endothelial Cell Viability

**Figure 5.**
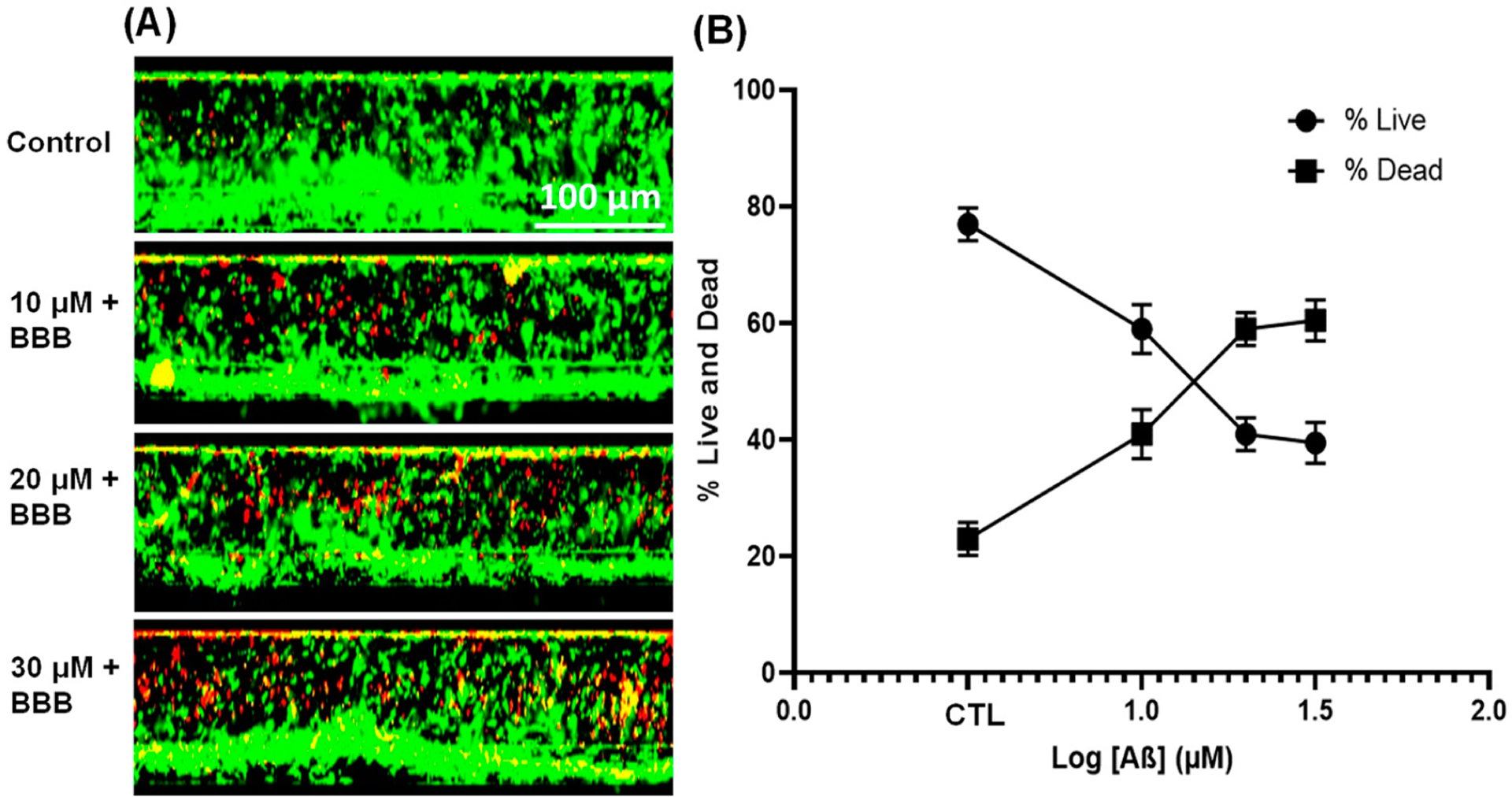
Cell viability assay of endothelial cells seeded on the two-lane microfluidics. (**A**) A representative series of confocal microscopy images depicting live/dead staining of endothelial tubules in microfluidic channels. Green fluorescence indicates live cells and red fluorescence indicates dead cells. (**B**) Percentage of live/dead 48 h following treatment with amyloid beta (Aβ_1–42_). All data represent average viability measured within the top and bottom layers of the endothelial tubule. The value represents the mean ± SD for two independent experiments (n = 2 for each treatment condition).

We quantified the effects of Aβ_1–42_ on endothelial cell viability after 48 h of incubation (Figure 5). The cell viability assessment using confocal images of live/dead assay demonstrated a dose-dependent decrease in endothelial cell viability, accompanied by significant cell death (Figure 5A,B), with an estimated IC50 within 5.29 ± 7. When compared with the BBB-No Aβ (CTL; 76.9%, *p* < 0.001), exposure of Aβ_1–42_ at 10 μM, 20 μM, and 30 μM to the blood lane significantly reduced the viability of endothelial cells by 59.1% (*p* = 0.0030), 40.8% (*p* = 0.0030), and 39.4% (*p* = 0.0011), respectively (Figure 5B). However, when the concentration gradient was compared to the viability, 20 μM and 30 μM were found to be comparable (*p* = 0.9698) but significantly lower than 10 μM (Figure 5, *p* = 0.0020).

## 4. Discussion

In this study, we present the creation of a high-throughput microfluidic device based on an in vitro model of the human blood–brain barrier (BBB) [21,29]. The patterning of ECM is made possible by the surface tension of the microfluidic platform. A BBB microvessel forms in the perfusion channel next to the ECM. The technique enables fluid-phase sampling of molecules that flow through the endothelium and extracellular matrix layers, allowing fluid to flow through BBB microvessels without the need for artificial membranes. We demonstrate that the model is sensitive to variations in Aβ concentrations. The focus of the paper is to investigate the transportation of Aβ oligomers across the blood–brain barrier (BBB), specifically examining Aβ accumulation and penetration at the BBB. This study has the potential to pave the way for future research to explore the role of Aβ transporters such as RAGE and LRP1 in mediating Aβ transport across the BBB. It is worth noting that the cells utilized in this model are immortalized, they may not have the same phenotype as BBB cells found in native cells. As a result, patient outcomes from this model might not be applicable directly. Nonetheless, regardless of the passage number, it has been demonstrated that the endothelial cell line in this investigation expresses tight junctional markers and forms BBB [30–34].

This paper examined the effect of Aβ_1–42_ oligomer on the BBB grown on a membrane-free microfluidics device. We investigated (1) the integrity of the BBB, (2) the expression of ZO-1 tight junctional protein, and (3) cell viability before and after exposure to Aβ_1–42_ solutions. In the microfluidic device, the endothelial layer formed the 3D tubular structure against the membrane-free ECM, which provides a physiologically relevant exposure route for Aβ_1–42_. To determine the barrier integrity of the BBB, FITC was infused into the BBB cultures within the microfluidic device. Time-lapse photography was used to track FITC penetration in the ECM lane, and the apparent permeability (***P***_*app*_) was estimated. The same method was applied to determine ***P***_*app*_ in chips that had or did not have an Aβ_1–42_-treated endothelial microvessel. Our findings suggest that FITC diffuses through the ECM to the same degree whether the barrier is absent or the BBB has been modified with Aβ. The results demonstrate the increase in FITC permeability across the BBB with the increase in Aβ_1–42_ concentrations (Figure 3).

**Figure 6.**
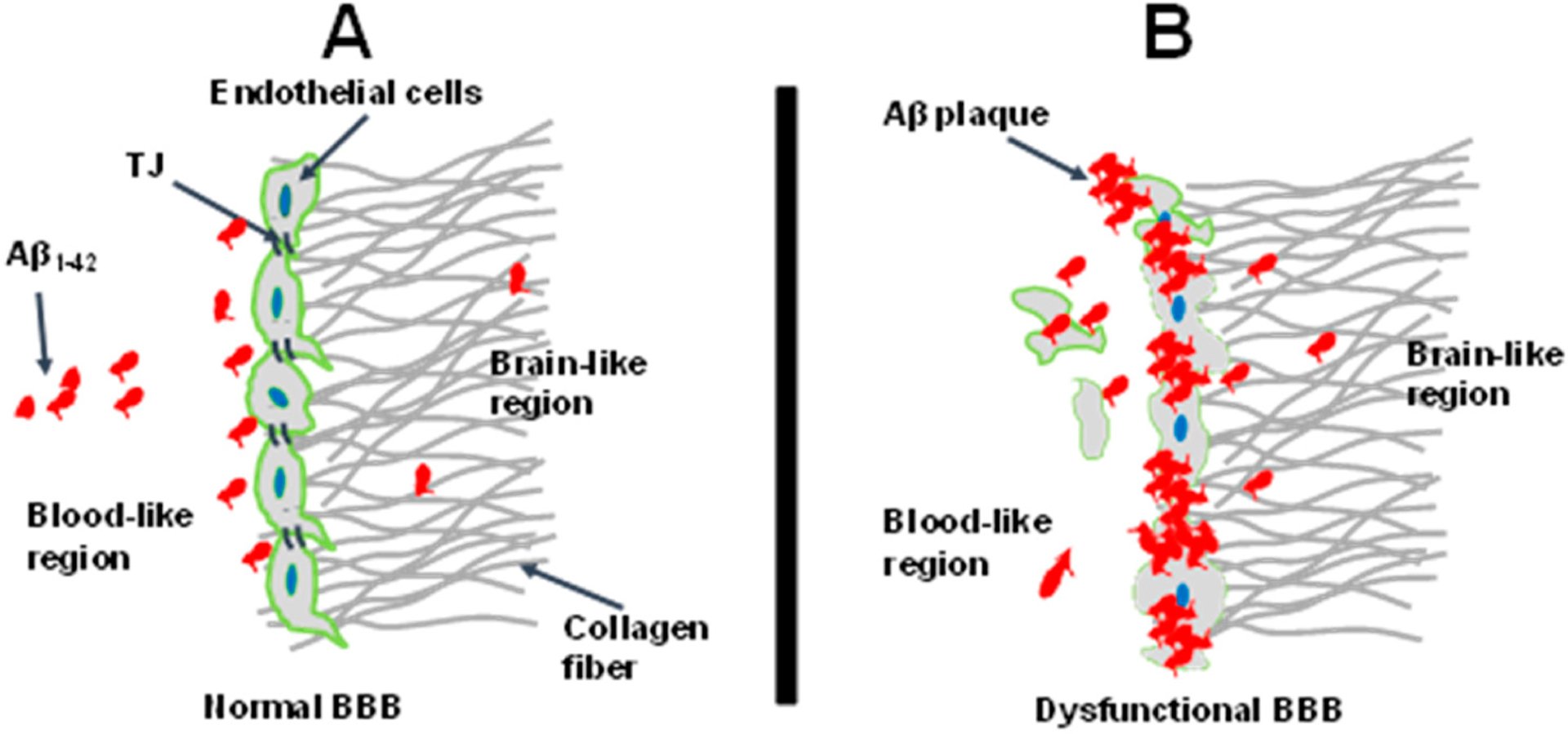
Schematic illustration of amyloid beta (Aβ) plaque aggregation with endothelial cells in the microfluidic device. Research rationale. (**A**): fully developed and functionally engineered BBB was challenged with different concentrations of Aβ_1–42_. (**B**): Aβ accumulates on the endothelial cells and forms cell–Aβ aggregates, eventually forming the thicker dysfunctional BBB. At this stage (**B**), the higher concentrations (20 μM and 30 μM) of Aβ produce a higher Aβ–cell aggregate that may diffuse into the brain region in a small amount. A low concentration such as 10 μM forms fewer aggregates with the cells, allowing individual oligomers to penetrate the barrier and reach the brain-like region. However, the Aβ–cells 3D complex structures at the barrier do not deter the permeability of FITC through the broken BBB and endothelial cells.

Interestingly, we observed that an increase in Aβ_1–42_ concentration was associated with an increase in the intensity of ZO-1 (Figure 4D,E). We believe that, when endothelial cells were treated with 20–30 μM Aβ_1–42_, Aβ was accumulated on the wall of ECs against the ECM and formed cell–Aβ matrix aggregates, eventually forming the aggregate of dysfunctional BBB (Figure 6). It seems as though the complex cell–Aβ matrix does not allow Aβ penetration into the brain parenchyma (ECM channel/brain lane), with the Aβ intensity in the ECM decreasing with increasing Aβ concentrations (Figure 4C). On the other hand, when endothelial cells were treated with a lower concentration such as 10 μM Aβ_1–42_, only a few locations showed cell–Aβ matrix aggregates bound, and cells maintained their homeostasis (Figure 4A,C). It is interesting to note that Aβ mimicked CAA pathogenesis by depositing on the wall of BBB against the ECM rather than passing through the compromised BBB into the brain lane. We believe that this will be the initial stage of Aβ accumulation on the BBB. Whether the aggregate of the cell–Aβ matrix complex structure will experience a reduction over time to allow the transport of Aβ_1–42_ into the brain region has not been investigated. Investigating if Aβ transporter expression occurs on the ECs of our model will be necessary for future research.

It has been reported that Aβ causes damage to TJs and directly impairs BBB integrity, confirming the relationship between Aβ and AD [35,36]. Using a transwell assay technique, human brain endothelial cells (hCMEC/D3) have been used as a BBB model and treated to 0–40 μM of Aβs. The concentration of Aβs has been shown to increase the penetration ability of FITC-dextran: 40 μM of Aβs has been shown to produce the highest permeability when compared to the control of 0 μM, while 5 μM has been found to produce a relatively small change [37]. We also confirmed that a low concentration of 10 μM and a higher concentration of 30 μM of Aβ_1–42_ for 48 h also induced structural damage in TJ (Figure 4A, *p* < 0.001). The FITC-dextran perfusion assay with microfluidic technology demonstrated increased perfusion with Aβ_1–42_ in a concentration-dependent manner (Figure 3, *p* < 0.001), suggesting that Aβ_1–42_ alters TJ architecture and digests BBB integrity [36,37]. Significant differences in the perfusion of FITC-dextran were found between the engineered BBB treated with Aβ_1–42_ and the control (untreated), highlighting a possible neurotoxic effect of Aβ_1–42_.

Of note is the fact that the concentrations of Aβ_1–42_ used in this study are higher than the physiological concentration of Aβ_40/42_ in the blood [38]. However, we did not observe any defined mechanism of Aβ_1–42_ localization. There is published evidence that when the endothelial cells are impaired they release proinflammatory cytokines, increasing neuroinflammation and secondary alteration, ultimately resulting in the disruption of the TJ and BBB [39]. The cell stretching due to accumulation of Aβ_1–42_ may be due to impaired cell cytoskeleton, retard movement of the endothelial cells, and cause their death [40]. Aβ affects endothelial cells (ECs) in the brain microvasculature directly or indirectly by changing TJP distribution, encouraging EC mortality, increasing oxidative stress, and stimulating glial cell production of pro-inflammatory cytokines [38,41]. In CAA mice models and human patients, Aβ buildup has a deleterious effect on brain artery walls, resulting in BBB disruption and possible bleeding [42,43]. Research conducted in vitro has demonstrated that exposure to Aβ leads to actin organization disruption and death in ECs [40]. Moreover, matrix metalloproteinases (MMPs) are expressed at higher levels while TJ protein expression is diminished in CAA patients and mice overexpressing amyloid precursor protein (APP) [43]. Additionally, the presence of Aβ inhibits the interaction between heat shock protein 90 and endothelial nitric oxide synthase [44] and encourages the development of von Willebrand factor (VWF) fibrils, which are linked to thrombogenic and inflammatory reactions in brain arteries [45]. These published results emphasize the harmful effects of Aβ deposition on artery walls, including the disruption of ECs and a weakened BBB. The loss of vascular control may be caused by any one of the above processes. We chose the most critical factors for in vitro modeling, including (1) endothelial cell barrier formation, (2) perfusion in a high throughput manner, and (3) membrane-free layer. This allowed the study of direct interactions between amyloid beta and the barrier layer such as accumulation of amyloid beta oligomer. Subsequent investigations with more complex vascular cells such as astrocytes and pericytes may provide better insights into amyloid beta interactions with the BBB.

## 5. Conclusions

We effectively created a three-dimensional perfused BBB-on-a-chip and studied Aβ trafficking. The resulting vascular network displayed lumens and BBB features. We examined the effect of Aβ_1–42_ on the physiological function of BBB in a perfusion channel. Our findings demonstrate a pronounced accumulation of Aβ_1–42_ oligomers at the BBB, resulting in the disruption of tight junctions and subsequent leakage evidenced by barrier integrity assay. We confirmed the formation of tubules in the microfluidic chips and that TJ plays an important role in BBB permeability.

## Data Availability

Data are contained within the article.
